# Long-term drug persistence and clinical outcomes of ustekinumab in Crohn’s disease: a multicenter real-world study from the Hokkaido phoenix cohort

**DOI:** 10.1093/crocol/otag059

**Published:** 2026-06-19

**Authors:** Takahiro Ito, Atsuo Maemoto, Nobuhiro Ueno, Mikihiro Fujiya, Toshifumi Ashida, Daisuke Hirayama, Yoshihiro Yokoyama, Satoshi Motoya, Masanao Nasuno, Hiroki Tanaka, Takehiko Katsurada, Hiroshi Nakase

**Affiliations:** Inflammatory Bowel Disease Center, Sapporo Higashi Tokushukai Hospital, Sapporo, 065-0033, Japan; Inflammatory Bowel Disease Center, Sapporo Higashi Tokushukai Hospital, Sapporo, 065-0033, Japan; Division of Gastroenterology, Department of Internal Medicine, Asahikawa Medical University, Asahikawa, 078-8510, Japan; Division of Gastroenterology, Department of Internal Medicine, Asahikawa Medical University, Asahikawa, 078-8510, Japan; Inflammatory Bowel Disease Center, Sapporo Tokushukai Hospital, Sapporo, 004-0041, Japan; Inflammatory Bowel Disease Center, Hakodate Goryoukaku Hospital, Hakodate, 040-8611, Japan; Division of Gastroenterology and Hepatology, Department of Internal Medicine, Sapporo Medical University School of Medicine, Sapporo, 060-8556, Japan; IBD Center, Sapporo Kosei General Hospital, Sapporo, 060-0033, Japan; Sapporo IBD Clinic, Sapporo, 064-0919, Japan; Sapporo IBD Clinic, Sapporo, 064-0919, Japan; Department of Gastroenterology and Hepatology, Graduate School of Medicine, Hokkaido University, Sapporo, 060-8648, Japan; Division of Gastroenterology and Hepatology, Department of Internal Medicine, Sapporo Medical University School of Medicine, Sapporo, 060-8556, Japan

**Keywords:** Crohn’s disease, drug persistence, long-term effectiveness, ustekinumab

## Abstract

**Background:**

Although ustekinumab (UST) is effective in Crohn’s disease (CD), long-term real-world data beyond 5 years and its effects on extraintestinal manifestations remain limited. We evaluated UST’s long-term effectiveness and safety in CD.

**Methods:**

This multicenter retrospective study enrolled 317 CD patients who initiated UST between May 2017 and February 2023 at seven institutions in Japan. The primary outcome was the UST continuation rate. The secondary outcomes included factors affecting the continuation rates, surgery-free survival, endoscopic remission rates, improvement in perianal and extraintestinal manifestations, and adverse events.

**Results:**

UST continuation rates at 1, 3, and 5 years were 87.6%, 69.6%, and 66.8%, respectively, with 43 patients followed beyond 5 years (maximum 6.1 years). Multivariate analysis identified week-8 albumin ≥3.3 g/dL (HR 0.30; 95% CI, 0.12-0.72; *P* = .007) and week-8 C-reactive protein (CRP) ≥0.33 mg/dL (HR 2.05; 95% CI, 1.14-3.69; *P* = .016) as independent predictors of continuation. Endoscopic remission rates improved significantly from baseline (10%) to week 52 (19%, *P* < .01) and week 104 (30%, *P* < .01). Among perianal fistula cases (*n* = 60), 28% achieved clinical fistula remission and 37% showed improvement. Resolution or improvement was observed in 76% of arthritis and 86% of psoriasis-like lesion cases. The surgery-free survival rate at 5 years was 85.1%. The adverse event rate was 10.7%.

**Conclusions:**

UST demonstrated favorable medium- to long-term effectiveness and safety in CD patients with limited follow-up data beyond 5 years. Week-8 albumin and CRP levels may serve as early predictive biomarkers but require external validation in independent cohorts.

## Graphical Abstract

**Graphical Abstract:**
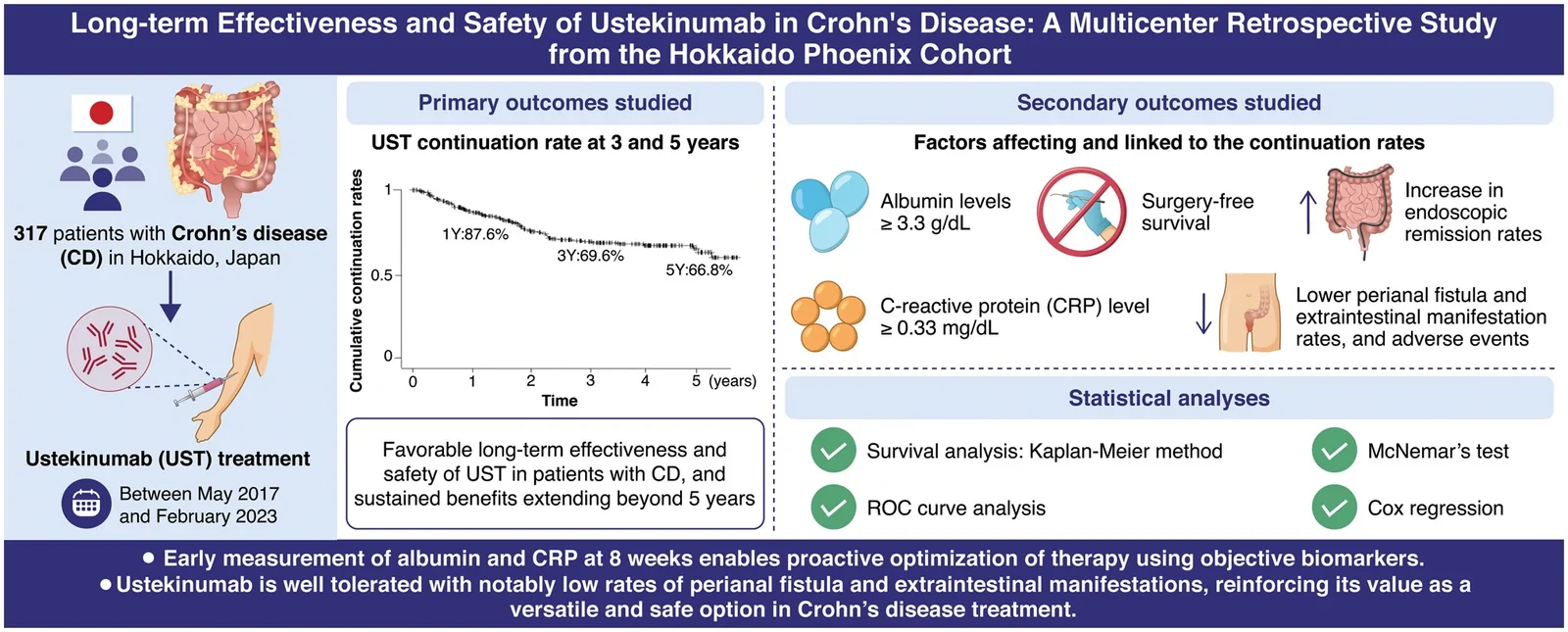

## Introduction

Following the demonstration of the efficacy of ustekinumab (UST) in pivotal phase 3 trials (UNITI-1, UNITI-2, and IM-UNITI),^1^ UST has been increasingly adopted in clinical practice worldwide. It offers an alternative mechanism of action to anti-tumor necrosis factor (TNF) therapies and gut-selective anti-integrin antibodies. UST is a fully human monoclonal antibody targeting the p40 subunit of interleukin (IL)-12 and -23 and represents a distinct therapeutic approach for Crohn’s disease (CD) by disrupting the IL-12/IL-23 signaling pathway crucial for T-helper cell differentiation and inflammatory responses.^1^

Since UST’s approval, real-world evidence has accumulated, with studies from various countries reporting its effectiveness in both bio-naïve and bio-experienced patients.^2^^,^^3^ In Japan, retrospective data for over a 3-year period post-marketing surveillance have confirmed its safety profile and effectiveness in Japanese populations.^4^^,^^5^ The present cohort partially overlaps with our previously published multicenter study;^5^ 137 of the 317 patients (43.2%) are shared. The current study expands the dataset by adding 16 patients from one additional institution and 164 patients enrolled after September 2019 and extends the observation period from a maximum of 2 years to a maximum of 6.1 years. The present cohort is independent from the nationwide post-marketing surveillance study.^4^ Many gaps still exist, particularly those related to UST’s long-term outcomes beyond 5 years, predictive biomarkers for response, and effectiveness in complex disease phenotypes, including perianal disease and extraintestinal manifestations.

The management of CD requires careful consideration of treatment durability, as the chronic inflammatory nature of the disease necessitates sustained therapeutic efficacy. Moreover, CD is frequently associated with complications beyond intestinal inflammation, including perianal fistulas affecting up to 40% of patients and extraintestinal manifestations in 25%-40% of cases.^6^ Understanding how UST performs across these diverse disease manifestations over extended periods of time is essential for optimizing the treatment algorithms and personalizing therapy selection.

Furthermore, with the recent approval of selective IL-23p19 and Janus kinase (JAK) inhibitors, the expanding therapeutic landscape for CD necessitates a better understanding of treatment sequencing and post-UST therapeutic options.^7^^,^^8^ Real-world data on the outcomes following UST discontinuation can improve clinical decision-making in the era of multiple advanced therapeutic options.

In the present study, we aimed to address these knowledge gaps by evaluating (1) the long-term effectiveness and safety of UST beyond 5 years in a real-world Japanese CD population, (2) the early predictive biomarkers for sustained treatment response, (3) UST’s effectiveness across various disease manifestations, including perianal and extraintestinal complications, and (4) the treatment outcomes following UST discontinuation to guide therapeutic sequencing decisions.

## Materials and methods

### Study design and patients

This multicenter retrospective observational study enrolled CD patients who initiated UST between May 2017 and February 2023 at 7 institutions in Japan. The study protocol was determined by the study group and approved by the ethics committee. The present study is registered at the University Hospital Medical Information Network Center before starting (UMIN, available at http://www.umin.ac.jp/ctr/). The present study was conducted according to the guidelines stipulated in the Declaration of Helsinki and adhered to the STROBE criteria for retrospective studies. Obtaining informed consent was waived owing to the retrospective nature of the study, and an opt-out approach was used in accordance with institutional ethical guidelines.

All patients were diagnosed with CD based on the criteria of the Japanese Ministry of Health, Labor and Welfare. These criteria are similar in scope to internationally recognized diagnostic criteria, with diagnosis based on a combination of clinical, endoscopic, histological, and radiological findings. There were no exclusion criteria. Patient data were collected from medical records using a standardized data collection approach across all centers; key variables including demographics, disease history, treatment history, concomitant medications, and treatment responses were recorded using a pre-specified data collection form.

### Outcomes

The primary outcome was the UST continuation rate. The secondary outcomes included factors affecting the continuation rates, cumulative surgery-free survival, endoscopic remission rates, improvement rates in perianal fistulas, changes in extraintestinal manifestations, adverse events, and post-UST treatment outcomes.

### Definitions

Disease activity was assessed using the Crohn’s Disease Activity Index (CDAI) and Simple Endoscopic Score for Crohn’s Disease (SES-CD).^9^ Endoscopic remission in our primary analysis was defined as the absence of all active inflammatory lesions (aphthae, erosions, and ulcers) within the observable range at ileocolonoscopy, corresponding to the “ulcer-related sub-score = 0” of the SES-CD across all examined ileocolonic segments. We did not use the composite SES-CD ≤2 cutoff for the primary endpoint because the SES-CD assigns 0-3 points for the presence of narrowings/stenoses irrespective of whether these are inflammatory or fibrotic in nature; in CD, fibrostenotic complications often persist after inflammatory healing, and a composite cutoff that incorporates the narrowing sub-score therefore systematically misclassifies patients with healed inflammation but residual fibrotic stricture as “not in remission.” The ulcer-absence criterion adopted here is consistent with the endoscopic-healing target endorsed by the STRIDE-II treat-to-target consensus of the International Organization for the Study of IBD.^10^

For perianal fistulas, “clinical fistula remission” was defined as complete cessation of drainage on clinical assessment by the treating physician documented in medical records; radiological confirmation of fistula closure (eg pelvic MRI, endoanal ultrasound) was not systematically performed and was therefore not used. “Improvement” was defined as a clinician-assessed reduction of approximately ≥50% in drainage activity compared with baseline; because drainage volume is not routinely quantified in clinical practice, this is a subjective assessment based on the treating physician’s clinical judgment documented in medical records.

Dose-interval modifications were made at the discretion of the treating physician, based on a combination of clinical symptoms, biomarker trends (CRP, albumin), endoscopic activity when available, and patient preference. Modifications consisted of interval shortening (12→8 weeks) for inadequate response or interval extension (8→12 weeks) in patients with sustained remission. Patients who underwent any dose-interval modification were treated as continuing UST until subsequent discontinuation or censoring.

### Statistical analysis

All statistical tests were conducted using EZR (Saitama Medical Center, Jichi Medical University, Saitama, Japan) graphical user interface for R (The R Foundation for Statistical Computing, Vienna, Austria). Categorical data were expressed as frequencies and percentages, whereas quantitative data were presented as medians and ranges (non-parametric data).

Kaplan–Meier method was used for the survival analysis. Time to discontinuation was defined as the number of days from UST initiation to the date of treatment cessation due to ineffectiveness or adverse events. Patients who were still receiving UST at the last observation date, those who transferred to other institutions, or those who self-discontinued were censored at their last documented follow-up date. Since Kaplan–Meier survival analysis appropriately handles variable follow-up durations through right-censoring, patients with shorter follow-up periods were not excluded from the analysis. The number of patients at risk at each time point is reported alongside all Kaplan–Meier curves.

To determine the optimal cutoff values of the predictors (eg disease duration, CDAI score, CRP, and albumin), we performed a receiver operating characteristic (ROC) curve analysis with maximum sensitivity and specificity for the continuation rates at week 52. Cutoff values for week-8 biomarkers identified by ROC analysis were internally validated by non-parametric bootstrap resampling (1000 iterations); the stability of the hazard-ratio estimates from the multivariate Cox model was likewise evaluated. The factors affecting the continuation rates were analyzed using Cox proportional hazards models. Univariate Cox regression was performed for all candidate clinical and biochemical predictors; variables with univariate log-rank *P* < .10 were eligible for the multivariate model. To minimize collinearity among complete blood count parameters, only platelet count was retained as the representative hematological marker, and the remaining acute-phase indicators selected were albumin and CRP, on the basis of their direct biological link to inflammatory and nutritional status. A pre-specified sensitivity model adding week-8 white blood cell (WBC) count was also performed and is reported. The changes in categorical variables were compared using McNemar’s test. *P*-value of <.05 were considered statistically significant.

## Results

### Patient characteristics

Altogether, 317 patients were included in this study, with 200 men (63.1%) and a median age of 38 years (range: 14-79 years). The median disease duration and follow-up period were 11 years (range: 0-43 years) and 118 weeks (range: 0-316 weeks, respectively).

Regarding disease location, 220 patients (69.4%) had ileocolonic disease (L3), whereas 75 (23.7%) had isolated ileal disease (L1) and 22 (6.9%) had isolated colonic disease (L2). The disease behaviors included stricturing disease (B2) in 138 patients (43.5%), inflammatory (B1) in 88 (27.8%), and penetrating (B3) in 91 (28.7%). Perianal fistulas were present in 60 patients (18.9%), and the extraintestinal manifestations included skin lesions in 37 patients (11.7%) and joint symptoms in 21 (6.6%).

### Treatment history and baseline characteristics

Among the patients, 252 (79.5%) had previous exposure to biological agents, with 107 (33.8%) using two or more biologics. The median baseline CDAI was 136 (range: 0-515), the median serum albumin level was 3.7 g/dL (range: 1.6-4.8 g/dL), and the median CRP was 0.3 mg/dL (range: 0.01-17.35 mg/dL). The median baseline SES-CD was 10 (range: 0-41).

The concurrent medications at the time of UST initiation included elemental nutrition (≥900 kcal/day) in 91 (28.7%), steroids in 120 (37.9%), and immunomodulators in 128 (40.4%) (Table 1). Among the 18 patients with a history of malignancy, the cancer types included colorectal cancer (*n* = 5), gastric cancer (*n* = 4), breast cancer (*n* = 3), lung cancer (*n* = 2), thyroid cancer (*n* = 2), and others (*n* = 2).

**Table 1:** Baseline characteristics of studied patients.

Characteristic (*N* = 317)	*n* (%) or median (range)
**Male**	200 (63.1)
**Age**	38 (14-79)
**Disease duration (years)**	11 (0-43)
**Observation period (weeks)**	118 (0-316)
**Disease location (L1 ileum: L2 colon: L3 ileocolon)**	75 (23.7): 22 (6.9): 220 (69.4)
**Disease behavior (B1 inflammatory: B2 stricturing: B3 penetrating)**	88 (27.8): 138 (43.5): 91 (28.7)
**Anal fistula**	60 (18.9)
**Skin manifestations**	37 (11.7)
**Joint symptoms**	21 (6.6)
**History of bowel resection (none: once : ≥2 times)**	156 (49.2): 82 (25.9): 79 (24.9)
**History of biologics use**	252 (79.5)
**History of ≥2 biologics**	107 (33.8)
**Vedolizumab only**	2 (0.6)
**History of malignancy (none: present: under treatment)**	299 (94.3): 14 (4.4): 4 (1.3)
**Disease activity**	
** CDAI**	136 (0-515)
** Albumin (g/dL)**	3.7 (1.6-4.8)
** CRP (mg/dL)**	0.3 (0.01-17.35)
** Endoscopic activity (SES-CD)**	10 (0-41)
**Concomitant medications at induction**	
** Elemental diet (≥900 kcal/day)**	91 (28.7)
** Steroids (prednisolone or budesonide)**	120 (37.9)
** Immunomodulators**	128 (40.4)
**UST dosing interval**	Every 8 weeks: 290 (91.5) Every 12 weeks: 5 (1.6) Interval changed: 22 (6.9)

The primary reasons for initiating UST were clinical symptoms (54%), endoscopic findings (25%), and adverse events from previous treatments (12%).

Regarding the UST dosing interval of subcutaneous injection, 290 patients (91.5%) received every-8-week dosing, 5 (1.6%) received every-12-week dosing, and 22 (6.9%) underwent a dosing interval change during follow-up; of these, 10 patients (3.2%) had their interval shortened from 12 to 8 weeks due to insufficient response, and 9 (2.8%) had their interval extended from 8 to 12 weeks, 2 (0.6%) underwent re-intensification (8→12→8 weeks), and 1 (0.3%) underwent shortening from 12 to 10 weeks. The dose-interval-change subgroup had 3-year UST persistence comparable to the non-modified group (∼73% vs. 70%; log-rank *P* = .78); detailed patterns and indications are provided in Table S1. Therapeutic drug monitoring of UST serum concentrations was not performed in any patient, as this is not available in routine clinical practice in Japan.

### UST continuation rate and its predictive factors

**Figure 1:**
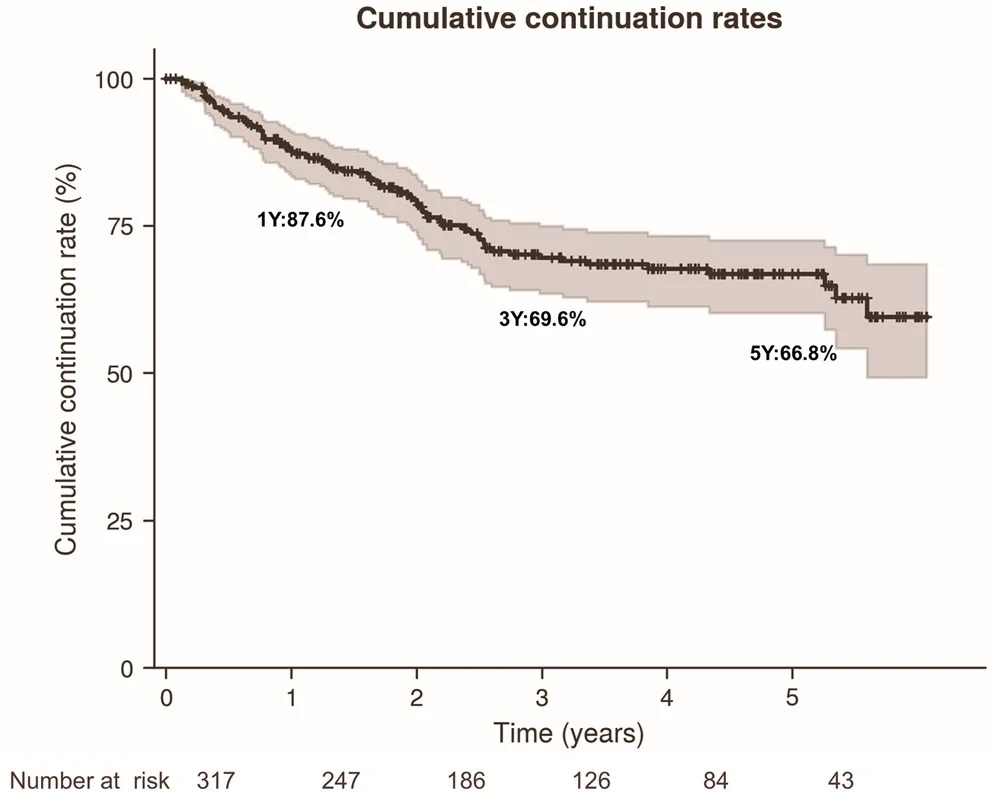
Cumulative UST continuation rates. The Kaplan–Meier curve shows the cumulative UST continuation rates over time. The continuation rates at 1, 2, 3, and 5 years were 87.6% (95% CI, 83.3-90.9), 79.0% (95% CI, 73.7-83.4), 69.6% (95% CI, 63.5-75.0), and 66.8% (95% CI, 60.2-72.5), respectively. The numbers of patients at risk at 0, 1, 2, 3, 4, and 5 years were 317, 247, 186, 126, 84, and 43, respectively. UST, ustekinumab.

The cumulative continuation rates for UST at 1, 2, 3, and 5 years were 87.6% (95% CI, 83.3-90.9), 79.0% (95% CI, 73.7-83.4), 69.6% (95% CI, 63.5-75.0), and 66.8% (95% CI, 60.2-72.5), respectively (Figure 1). Regarding follow-up beyond 5 years, 43 patients (14%) had follow-up data exceeding 5 years, with 4 patients followed beyond 6 years and a maximum observation period of 315.7 weeks (6.1 years). All 120 patients who were receiving steroids at the start of the UST discontinued steroid use within 1 year.

In the univariate analysis, the following baseline parameters were significantly associated with UST continuation: body mass index (BMI) ≥19.8 kg/m^2^ (*P* = .043), skin lesions excluding atopic dermatitis (*P* = .040), albumin level of ≥3.1 g/dL (*P* = .018), CRP level of ≥0.15 mg/dL (*P* = .036), and platelet count of ≥33.4 × 10^4^/μL (*P* = .049). Moreover, the following week-8 parameters demonstrated significant associations with UST continuation: white blood cell count of ≥6660/μL (*P* = .003), platelet count of ≥32.4 × 10^4^/μL (*P* = .034), albumin level of ≥3.3 g/dL (*P* < .001), CRP level of ≥0.33 mg/dL (*P* < .001), and achievement of clinical remission (*P* = .001) (Figure S1).

In the multivariate analysis, only the week-8 albumin level of ≥3.3 g/dL (hazard ratio [HR], 0.30; 95% confidence interval [95%CI], 0.12-0.72, *P* = .007) was significantly associated with better continuation rates, whereas the week-8 CRP level of ≥0.33 mg/dL (HR, 2.05; 95% CI, 1.14-3.69; *P* =.016) was significantly associated with lower continuation rates. Other clinical factors, including BMI of ≥19.8 kg/m^2^ (HR, 0.68; 95% CI, 0.40-1.1; *P* =.156) and baseline albumin level of ≥3.1 g/dL (HR, 2.02; 95% CI, 0.82-4.95; *P* = .125) showed trends, but they did not reach statistical significance in the multivariate model (Table 2). In a pre-specified sensitivity model that additionally entered week-8 WBC ≥6,660/μL into the multivariate Cox model, the original biomarkers retained statistical significance (week-8 albumin ≥3.3 g/dL: HR 0.31, 95% CI, 0.13-0.75, *P* =.009; week-8 CRP ≥0.33 mg/dL: HR 1.87, 95% CI, 1.03-3.37, *P* = .039) and week-8 WBC ≥6,660/μL was an independent predictor (HR 1.84, 95% CI, 1.06-3.20, *P* = .030).

**Table 2:** Univariate and multivariate regression analyses for identifying the predictors of continuation of UST.

			Univariate	Multivariate		
			*P* *	HR	95%CI	*P* **
**Age ≥35 years at inclusion**			.062			
**Sex**			.326			
**Disease duration, ≥224 months**			.226			
**BMI ≥ 19.8**			.043	0.68	0.40-1.16	.156
**Disease location**	L1 (Ileum)		.108			
**Disease behavior**	B1 (Inflammatory disease)		.162			
	B3 (Penetrating disease)		.897			
**Anal lesions**	Any lesions		.054	1.27	0.77-2.11	.355
	Anal fistula		.205			
**Skin lesion (excluding atopic dermatitis)**			.040	0.33	0.08-1.37	.125
**Smoking history (never, past, current)**			.120			
**Prior intestinal resections**			.144			
**Prior biologics**	Naive to biologics		.740			
	2 or more biologics		.122			
**Disease activity**	baseline	CDAI ≥ 220	.070			
		WBC ≥ 7600/µL	.093			
		Hb ≥ 10.8 g/dL	.427			
		Plt ≥ 33.4 × 10^4^/µL	.049			
		Albumin ≥ 3.1 g/dL	.018	2.02	0.82-4.95	.125
		CRP ≥ 0.15 mg/dL	.036	0.93	0.48-1.80	.825
		SES-CD ≥ 10	.176			
	week 8	WBC ≥ 6660/µL	.003			
		Hb ≥ 12.0 g/dL	.114			
		Plt ≥ 32.4 × 10^4^/µL	.034	1.66	0.99-2.80	.067
		Albumin ≥ 3.3 g/dL	<.001	0.30	0.12-0.72	.007
		ΔAlbumin ≥ 0.2 g/dL	.318			
		CRP ≥ 0.33 mg/dL	<.001	2.05	1.14-3.69	.016
		Clinical remission	.001	0.79	0.43-1.45	.450
**Concomitant medication**	Enteral nutrition (≥900 kcal/day)		.919			
	Corticosteroids or budesonide		.442			
	Corticosteroids or budesonide at the induction phase		.222			
	Immunomodulators (thiopurines)		.492			

* Log-rank test.

** Cox proportional hazards regression analysis.

Internal validation of the ROC-derived week-8 cutoffs by 1000-iteration bootstrap resampling confirmed that the albumin cutoff was stable (median 3.25 g/dL; 86.0% of replicates within 3.0-3.6 g/dL) and the predictor directionality was preserved in 99.9% of replicates for albumin (HR < 1) and 99.4% for CRP (HR > 1). The CRP cutoff showed wider variability across replicates (median 0.41 mg/dL; 95% CI, 0.33-1.32).

Of the 317 patients, 17 (5.4%) initiated UST primarily for post-operative remission maintenance (“post-surgical reset”). In this subgroup, the 1-, 3-, and 5-year continuation rates were 100%, 93.3%, and 93.3%, respectively (Figure S2). However, this subgroup had substantially lower baseline disease activity than the overall cohort (median baseline CDAI 107 vs. 136; CRP 0.18 vs. 0.31 mg/dL); only one patient had an active perianal fistula, and 16/17 had a history of bowel resection (Table S2). The high persistence rate in this subgroup therefore likely reflects the reduced inflammatory burden after recent surgery and selection bias toward patients with quiescent disease, rather than a differential therapeutic effect of UST.

### Effect of concomitant therapies

To examine whether concomitant therapies influenced UST persistence, we performed stratified Kaplan–Meier analyses (Table S3). The 5-year UST persistence rates did not differ significantly by concomitant steroid (60.6% vs. 70.8%, log-rank *P* = .22), immunomodulator (69.4% vs. 65.3%, *P* = .49), or enteral nutrition ≥900 kcal (63.4% vs. 68.3%, *P* = .92) status at induction. As all 120 patients on steroids at induction discontinued steroid therapy within the first year, the long-term continuation rates can be interpreted as steroid-free UST persistence.

### Surgery-free survival

**Figure 2:**
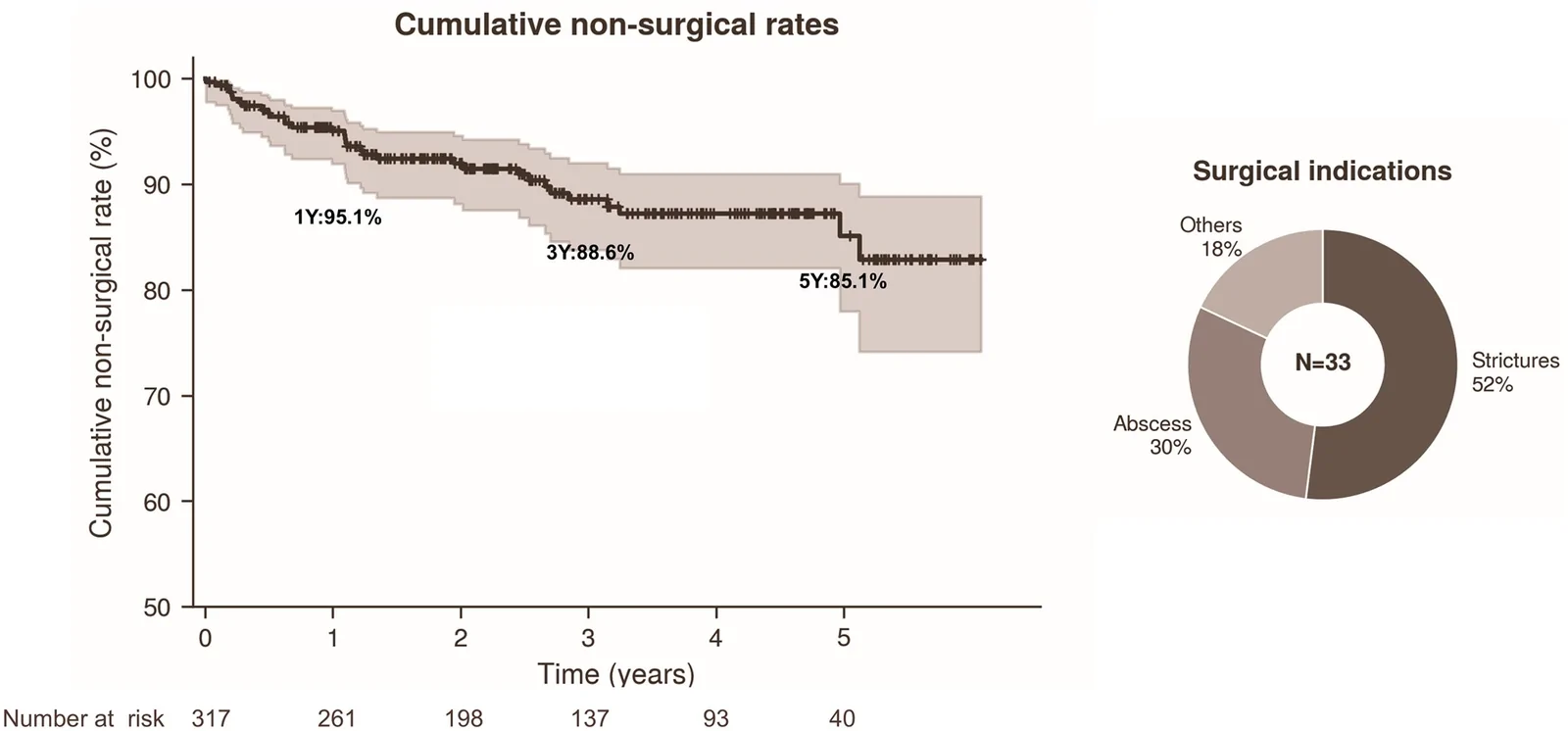
Cumulative non-surgical rates. The Kaplan–Meier curve shows the surgery-free survival rates over time. The non-surgical rates at 1, 2, 3, and 5 years were 95.1% (95% CI, 91.9-97.0), 92.0% (95% CI, 88.1-94.6), 88.6% (95% CI, 83.8-92.0), and 85.1% (95% CI, 78.0-90.1), respectively. The numbers of patients at risk at 0, 1, 2, 3, 4, and 5 years were 317, 261, 198, 137, 93, and 40, respectively. Among 33 patients requiring surgery during follow-up, the main indications were strictures (52%) and abscesses (30%), with 90% of surgeries occurring within 2 years of UST initiation. UST, ustekinumab.

The cumulative non-surgical rates at 1, 2, 3, and 5 years were 95.1% (95% CI, 91.9-97.0), 92.0% (95% CI, 88.1-94.6), 88.6% (95% CI, 83.8-92.0), and 85.1% (95% CI, 78.0-90.1), respectively (Figure 2). Among 33 patients requiring surgery during follow-up, the main indications were strictures (52%) and abscesses (30%), with 90% of surgeries occurring within 2 years of UST initiation.

### Endoscopic outcomes

**Figure 3:**
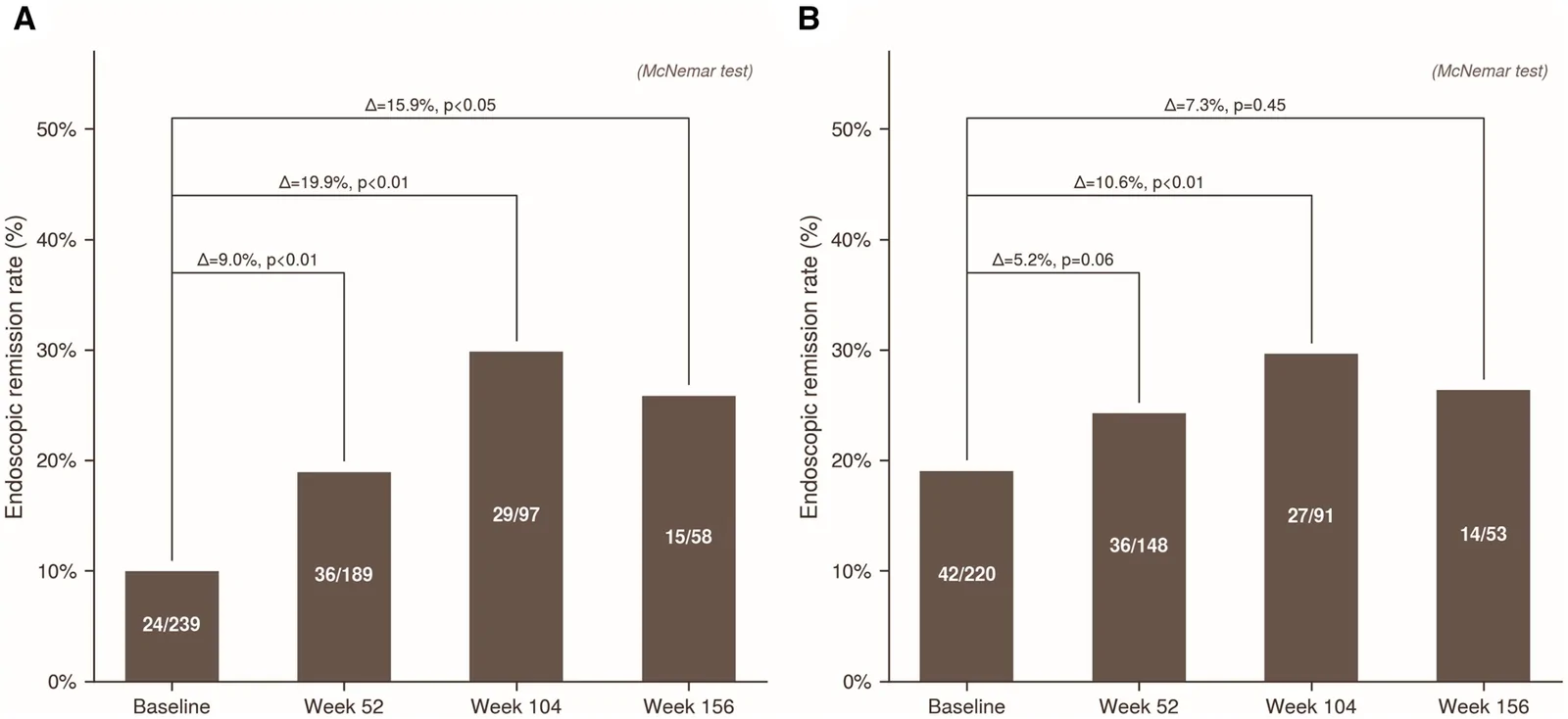
**Endoscopic remission rates**. (A) The overall endoscopic remission rates significantly improved from baseline (10%) to weeks 52 (19%, *P* <.01) and 104 (30%, *P* <.01). The denominator (number of patients with available endoscopy) at each time point is indicated in the figure. (B) For ileal lesions specifically, the remission rates improved from baseline (19%) to week 52 (24%, *P* = .06) and 104 (30%, *P* <.01).

The endoscopic remission rates showed significant improvement from baseline (10.0%) to weeks 52 (19.0%, *P* < .01) and 104 (29.9%, *P* < .01) (Figure 3A). For ileal lesions, the remission rates improved from baseline to weeks 52 (from 19.1% to 24.3%, *P* = .06) and 104 (from 19.1% to 29.7%, *P* < .01) (Figure 3B).

Among patients with available SES-CD data, the median SES-CD score decreased from 10 (range: 0-41) at baseline to 7 (range: 0-28) at week 52, 5 (range: 0-25) at week 104, and 4 (range: 0-22) at week 156, demonstrating a progressive reduction in endoscopic disease burden over time. The standardized SES-CD ≤2 sensitivity analysis showed broadly concordant rates with our primary ulcer-absence definition (10.6%, 16.2%, 25.3%, and 22.4% at baseline, week 52, week 104, and week 156, respectively; overall agreement 85.4%-95.7%, Cohen’s κ 0.52-0.80; Table S4). Endoscopic assessment beyond week 156 was limited by the smaller number of patients remaining on treatment with available follow-up endoscopy, which represents a limitation of the study.

### Perianal and extraintestinal manifestations

Among the 60 patients with perianal fistulas (44 with complex fistulas), 17/60 (28%) achieved clinical fistula remission (complete cessation of drainage), 22/60 (37%) showed improvement, 13/60 (22%) remained unchanged, and 8/60 (13%) worsened during a median follow-up of 118 weeks. In biologics-naïve patients (*n* = 18, 15 with complex fistulas), the outcomes were more favorable, with 9/18 (50%) achieving clinical fistula remission. Furthermore, among the 257 patients without fistulas at UST initiation, only three patients developed fistulas during follow-up.

**Figure 4:**
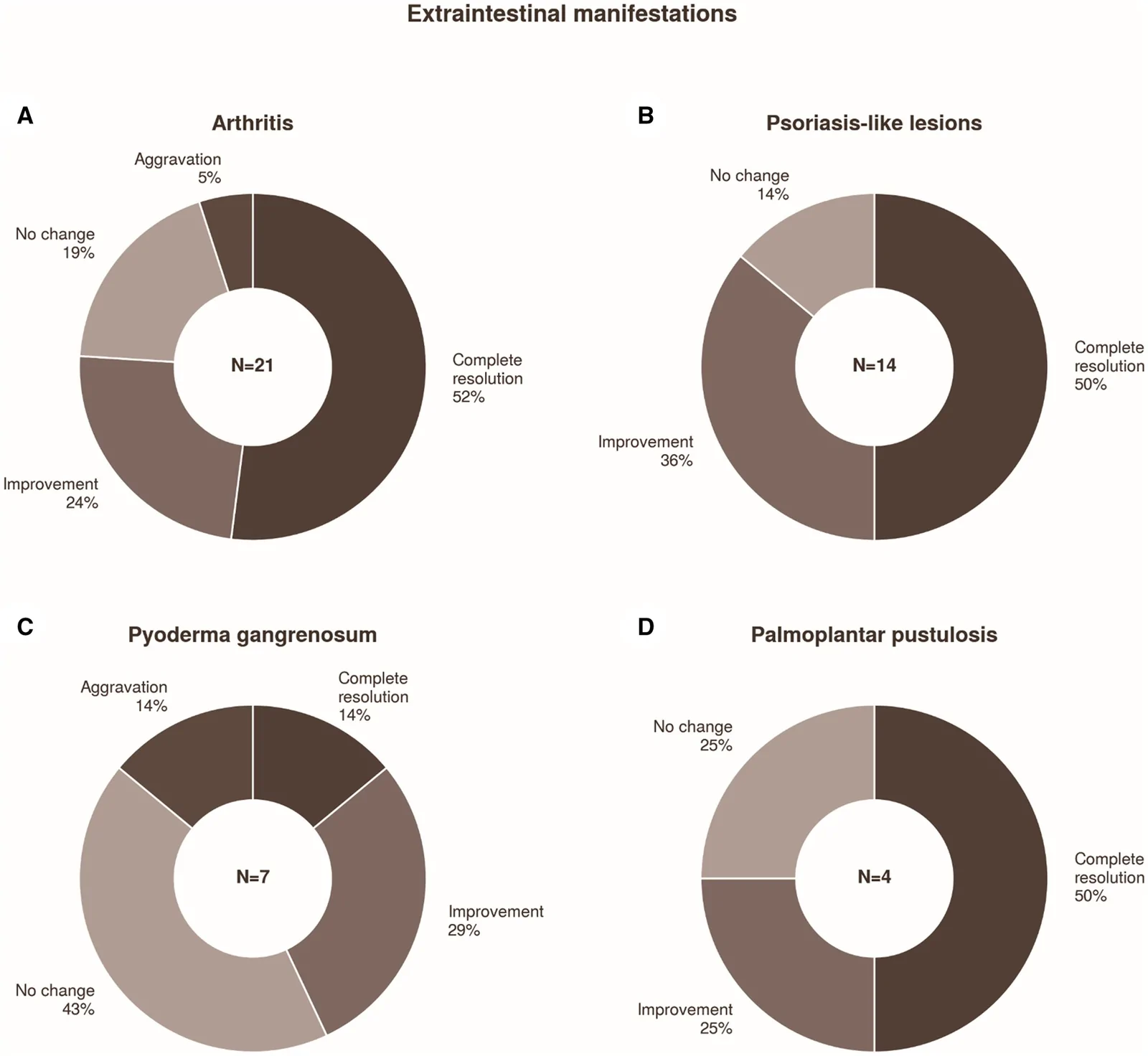
Extraintestinal manifestations. (A) Among patients with arthritis (*n* = 21), 11 (52%) achieved complete resolution and 5 (24%) showed improvement. (B) Among patients with psoriasis-like lesions (*n* = 14), 7 (50%) achieved complete resolution and 5 (36%) showed improvement. (C) Among patients with pyoderma gangrenosum (*n* = 7), 1 (14%) achieved complete resolution and 2 (29%) showed improvement. (D) Among patients with palmoplantar pustulosis (*n* = 4), 2 (50%) achieved complete resolution and 1 (25%) showed improvement.

Regarding the extraintestinal manifestations, among patients with arthritis (*n* = 21), 11/21 (52%) achieved complete resolution and 5/21 (24%) showed improvement (Figure 4A). For those with psoriasis-like lesions (*n* = 14), 7/14 (50%) achieved complete resolution and 5/14 (36%) showed improvement (Figure 4B). Among patients with pyoderma gangrenosum (*n* = 7), 1/7 (14%) achieved complete resolution and 2/7 (29%) showed improvement (Figure 4C). Among patients with palmoplantar pustulosis (*n* = 4), 2/4 (50%) achieved complete resolution and 1/4 (25%) showed improvement (Figure 4D). These extraintestinal-manifestation subgroup analyses involve small numbers and should be regarded as exploratory.

### Safety profile

Adverse events occurred in 34 patients (10.7%) during the median follow-up period of 118 weeks. The common adverse events included injection site reactions (*n* = 8), general fatigue (*n* = 8), and infections (*n* = 7) (Supplementary Table S5). Two cases of newly diagnosed malignancy occurred in patients without a prior history of malignancy; no recurrence or exacerbation was observed in the 18 patients with a prior cancer history. Only two patients discontinued treatment due to adverse events.

### UST discontinuation

**Figure 5:**
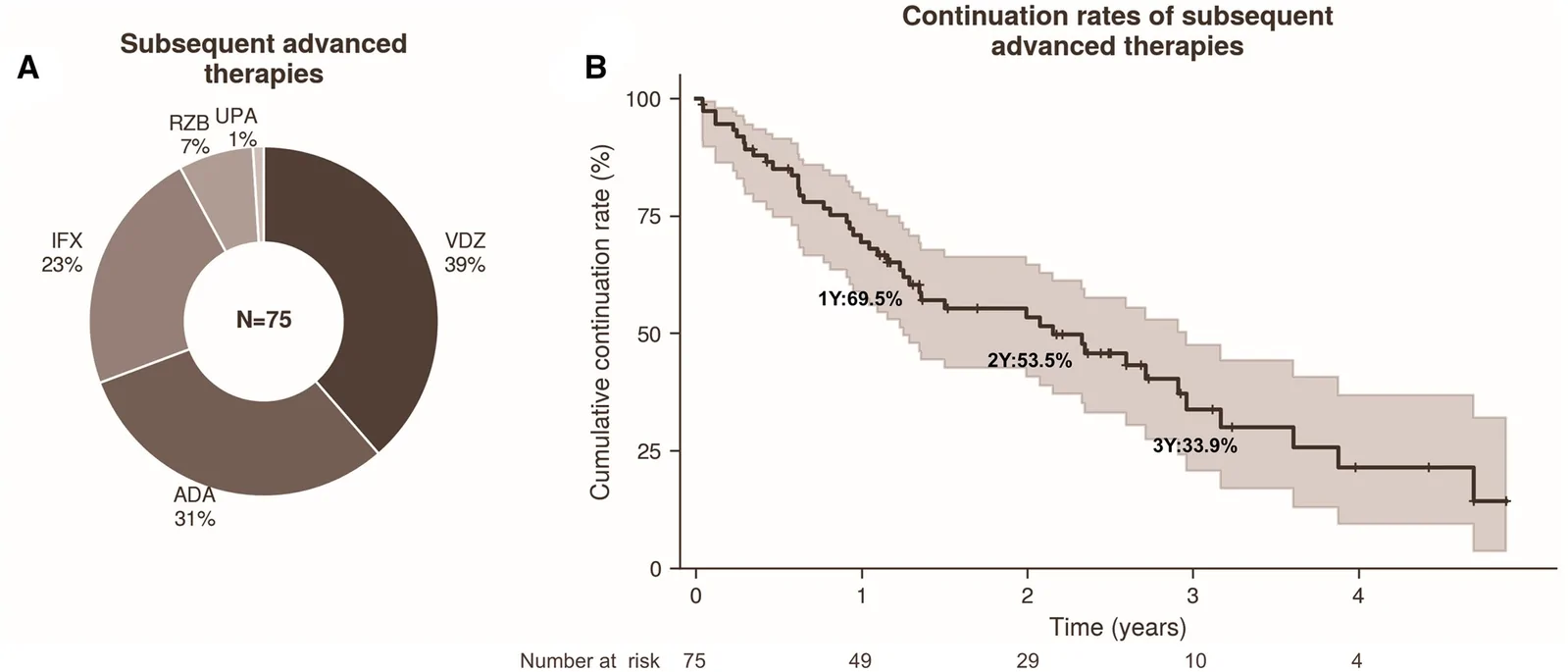
Post-UST discontinuation outcomes. (A) Among the 86 patients who discontinued UST, 75 received a subsequent advanced therapy (excluding patients who received no treatment, enrolled in clinical trials, or switched to other therapies). Subsequent treatments included vedolizumab (VDZ, 39%), adalimumab (ADA, 31%), infliximab (IFX, 23%), risankizumab (RZB, 7%), and upadacitinib (UPA, 1%). (B) The cumulative continuation rates for subsequent advanced therapies at 1, 2, and 3 years were 69.5% (95% CI, 57.2-78.6), 53.5% (95% CI, 40.6-64.4), and 33.9% (95% CI, 20.7-47.5), respectively. The numbers of patients at risk at 0, 1, 2, 3, and 4 years were 75, 49, 29, 10, and 4, respectively. UST, ustekinumab.

Altogether, 86 patients discontinued the UST treatment, with 84 discontinuing due to the ineffectiveness of UST and 2 discontinuing due to adverse events. Of the 86 patients who discontinued UST, 75 received a subsequent advanced therapy (vedolizumab, adalimumab, infliximab, risankizumab, or upadacitinib); patients who received no subsequent treatment, were enrolled in clinical trials, or switched to other non-advanced therapies were excluded from this analysis. The subsequent treatments included vedolizumab (VDZ, 39%), adalimumab (ADA, 31%), infliximab (IFX, 23%), risankizumab (RZB, 7%), and upadacitinib (UPA, 1%) (Figure 5A). The cumulative continuation rates for subsequent advanced therapies at 1, 2, and 3 years were 69.5% (95% CI, 57.2-78.6), 53.5% (95% CI, 40.6-64.4), and 33.9% (95% CI, 20.7-47.5), respectively (Figure 5B). Drug-class–stratified continuation rates of subsequent advanced therapies are presented in Figure S3 and Table S6. The 3-year continuation rates were 27.9% for anti-TNF agents (*n* = 40, IFX + ADA), 30.6% for vedolizumab (*n* = 29), and 83.3% for the small IL-23p19/JAK inhibitor subgroup (*n* = 6); these results are exploratory and limited by the sample size in each drug-class arm.

## Discussion

This multicenter retrospective study provides comprehensive real-world evidence for UST’s long-term effectiveness and safety in Japanese CD patients, addressing several critical knowledge gaps in the current literature. Our findings demonstrated sustained therapeutic benefits of UST beyond 5 years and identified the early predictive biomarkers. Our study provides novel insights into the treatment outcomes across diverse disease manifestations and post-UST-discontinuation therapeutic options.

The 5-year UST continuation rate of 67% observed in our cohort is consistent with and extends the real-world evidence reported from European cohorts. In the nationwide Finnish real-world study by Af Björkesten et al., drug persistence with UST was reported over a median follow-up of approximately 2 years.^11^ The Stockholm UST study by Bello et al. reported long-term real-world data with a similar follow-up profile.^12^ Additionally, the 5-year efficacy data from the IM-UNITI extension trial demonstrated continuation rates consistent with our findings.^13^ The comparison of our 5-year data with key published studies is summarized in Table 3. Notably, our cohort includes 43 patients following beyond 5 years (maximum 6.1 years), providing one of the longest real-world observation periods reported for UST in CD. The continued plateau in the continuation rate after 3 years (69.6% at 3 years vs. 66.8% at 5 years) suggests durable long-term maintenance of response in patients who remain on therapy, although the wide confidence intervals at 5 years (95% CI, 60.2-72.5) and the relatively small at-risk population beyond 5 years (*n* = 43) should be borne in mind when interpreting this point estimate. This sustained effectiveness is noteworthy given that 80% of our patients had prior biological exposures, with 34% experiencing treatment failure from two or more biologics, representing a treatment-refractory population.

**Table 3:** Comparison of 5-year UST continuation rates with published real-world studies.

Study	*n*	Follow-up	5-year rate	Population
**Present study**	317	Long-term Max 6.1 yr Median 27 mo	66.8%	Japanese real-world, multicenter
**IM-UNITI extension (Sandborn 2022)** ^13^	217	5 yr (RCT)	∼60%	International RCT
**Af Björkesten (2020)** ^11^	155	Max 2 yr Median 14 mo	N/A	Finnish real-world, multicenter
**Bello (2024)** ^12^	322	Long-term Max 3 yr Median 13 mo	N/A	Swedish real-world, multicenter
**Barkai (2024)** ^21^	148	Long-term Max 3 yr Median 38 mo	N/A	Hungarian real-world, multicenter
**Bressler (2023)** ^22^	8,724	Long-term Max 4 yr Median N/A	N/A	Canadian real-world, national cohort

Abbreviations: N/A, specific 5-year rate not separately reported. RCT, randomized controlled trial.

The durability of UST response in our cohort may reflect its unique mechanism of action. By targeting the shared p40 subunit of IL-12 and IL-23, UST disrupts both the Th1 and Th17 inflammatory pathways implicated in CD pathogenesis.^14^ This dual inhibition may provide more comprehensive inflammatory control as compared to therapies targeting single cytokines or pathways, potentially explaining the sustained long-term effectiveness observed.

One of the most important findings from our study is the identification of the week-8 biomarkers as predictors of long-term treatment success. The strong association between the albumin level of ≥3.3 g/dL at week 8 and improved continuation rates (HR, 0.30, *P* = .007), along with the negative predictive value of the elevated week-8 CRP level of ≥0.33 mg/dL (HR 2.05, *P* = .016), provides a practical framework for early treatment optimization. These cutoffs were derived from ROC analysis targeting week-52 continuation in the same cohort and should therefore be regarded as exploratory.

Internal validation by bootstrap resampling supported the stability of the albumin cutoff but revealed wider variability for the CRP threshold (Methods and Results); the 0.33 mg/dL cutoff should therefore be interpreted as one operational point within a broader “low-CRP” window. External validation in an independent cohort remains warranted before clinical implementation.

The timing for this evaluation is 8 weeks after the administration of the first dose of UST. For patients whose inflammatory markers have not normalized by week 8, dose optimization, including shortening the dosing interval (every 4-6 weeks) or re-dosing, may be effective,^15^^,^^16^ but dosing periods shorter than 8 weeks have not been approved in Japan. In the present study, the biomarkers at 8 weeks post-treatment are more prognostic than the biomarkers at the start of treatment, suggesting that the initial response trajectory, rather than disease severity at treatment initiation, determines the long-term success. This finding has important implications for patient counseling and shared decision-making, as it suggests that even patients with severe baseline disease can achieve durable remission if they demonstrate an early biochemical improvement.

In the current study, we also investigated the effectiveness of UST in managing the complications that CD patients experience. The observed perianal fistula outcomes, with 28% of the patients achieving complete closure and 37% showing improvement, are consistent with the results of the pooled analyses of the UNITI trials^17^ and with the BioLAP multicenter study from the GETAID, which reported clinical fistula response rates of similar magnitude in perianal CD patients treated with UST.^18^ These observations extend over a longer follow-up period than most previously published data. It should be noted, however, that our perianal fistula outcomes were assessed solely by clinical judgment of drainage activity documented in medical records, without systematic radiologic confirmation by pelvic MRI or endoanal ultrasound. Drainage volume was not objectively quantified, and the “≥50% reduction in drainage activity” used to define improvement was based on the treating physician’s subjective assessment. These methodological limitations may overestimate or underestimate the true fistula response rate, since clinical cessation of drainage does not necessarily correspond to structural fistula closure. Our results should therefore be interpreted with caution and viewed as an estimate of clinical, rather than radiologic, response. Future prospective studies incorporating MRI-based radiologic healing or standardized measures (eg the Fistula Drainage Assessment) are warranted. Importantly, biologics-naïve patients achieved a 50% clinical remission rate, suggesting that UST could be effective as the first-line biological therapy for not only simple fistulas but also complex fistulas. The high response rates for extraintestinal manifestations—76% for arthritis (16/21) and 86% for psoriasis-like lesions (12/14)—highlight UST’s systemic anti-inflammatory effects. These findings are particularly relevant, given the shared IL-23/Th17 pathway involvement in both intestinal and extraintestinal inflammations.^19^ The effectiveness across multiple disease domains positions UST as a comprehensive therapeutic option for patients with complex CD phenotypes, potentially reducing the need for additional systemic therapies for extraintestinal manifestations.

The progressive improvement in the endoscopic remission rates from 10% at baseline to 29.9% at week 104 demonstrates UST’s ability to achieve mucosal healing, which is increasingly recognized as the optimal therapeutic target in CD.^20^ The continued improvement between weeks 52 and 104 suggests that the maximal effect of UST on mucosal healing may require extended treatment duration, supporting the importance of maintaining therapy even in patients with an initial partial response. Our primary endoscopic-remission endpoint—the absence of all active inflammatory lesions (aphthae, erosions, and ulcers), corresponding to the ulcer-related sub-score = 0 of the SES-CD^9^—was deliberately chosen over the composite SES-CD ≤2 cutoff because the SES-CD assigns 0-3 points for the presence of narrowings/stenoses irrespective of whether these are inflammatory or fibrotic; in CD, fibrostenotic complications often persist after inflammatory healing, and a composite cutoff systematically misclassifies patients with healed inflammation but residual fibrotic stricture. The ulcer-absence criterion adopted here is consistent with the endoscopic-healing target endorsed by the STRIDE-II treat-to-target consensus.^10^ The SES-CD ≤2 sensitivity analysis (Table S4) yielded broadly concordant results (Cohen’s κ 0.52-0.80), with the residual numerical gap (eg 21.5% vs. 16.2% at week 52) concentrated in patients with healed inflammation but residual fibrotic stricture, supporting the rationale for our primary definition. Endoscopic follow-up beyond week 156 was limited by the decreasing number of patients with available endoscopic data, which is a recognized limitation of long-term retrospective real-world studies.

The favorable safety profile observed, with adverse events occurring in only 10.7% of patients over a median follow-up of 118 weeks (range 0-316 weeks), provides reassurance for the long-term use of UST. The low discontinuation rate due to adverse events (0.6%) and absence of malignancy progression in patients with prior cancer history align with the results of the integrated safety analyses of previous clinical trials^13^ and with the post-marketing surveillance data.^4^ This safety profile is particularly relevant for the management of CD, where the chronic-disease nature necessitates long-term treatment exposure.

Our analysis of the post-UST discontinuation outcomes addresses a critical knowledge gap in the era of expanding CD therapeutics. The observation that subsequent advanced therapies had lower continuation rates (34% at 3 years) than UST highlights the challenge of managing patients who have not responded to drugs with multiple mechanisms of action. In drug-class–stratified analyses (Table S6, Figure S3), 3-year continuation was 27.9% for anti-TNF agents, 30.6% for vedolizumab, and 83.3% for the small IL-23p19/JAK inhibitor subgroup, although small sample sizes preclude firm conclusions. The emergence of IL-23p19 selective^7^ and JAK^8^ inhibitors offers new options for UST-refractory patients, although optimal sequencing remains to be determined. Compared to the Stockholm UST study,^12^ our post-UST continuation rates were lower, which likely reflects the higher proportion of biologic-experienced patients in our cohort.

### Limitations

Several limitations merit consideration. First, the retrospective design introduces potential selection bias and limits the standardization of outcome assessments across centers. Although a pre-specified data collection form was used, we cannot rule out variability in clinical assessment. Second, the present cohort partially overlaps with our previously published multicenter study;^5^ specifically, 137 of the 317 patients (43.2%) overlap with our 2020 publication, while the remaining 180 patients (16 from a newly added 7th institution and 164 newly enrolled after September 2019) are reported here for the first time. To address this concern, we performed a sensitivity analysis stratified by enrollment window: the 1-, 3-, and 5-year UST continuation rates in the post-October 2019 (new only) subgroup were 87.8%, 63.2%, and 63.2%, respectively, with overlapping 95% confidence intervals to those of the full cohort and a non-significant log-rank test between subgroups (*P* =.34); the week-8 biomarker predictors retained statistical significance in this independent subgroup (Table S7). Third, the number of patients at risk declined substantially at later time points (*n* = 43 at 5 years), and confidence intervals accordingly widened; the long-term continuation rate estimates should therefore be interpreted with caution. Fourth, the censoring approach included patients who transferred to other institutions (*n* = 15) and those who self-discontinued (*n* = 4); the reasons for these were not systematically available, which may introduce informative censoring bias. Fifth, perianal fistula outcomes were assessed by clinical judgment without radiological confirmation of fistula closure, and drainage volume was not objectively quantified; these definitions may therefore underestimate or overestimate the true fistula response rate. Sixth, the endoscopic remission analysis was based on a binary endpoint, and follow-up SES-CD data were limited beyond week 156, restricting conclusions about long-term endoscopic effectiveness. Seventh, the ROC-derived biomarker cutoffs were derived post-hoc from the same cohort and require external validation. Finally, therapeutic drug monitoring of UST serum concentrations was not performed in any patient, as this is not available in routine clinical practice in Japan; this precludes the analysis of exposure–response relationships and the evaluation of whether sub-therapeutic drug levels contributed to treatment failure.

## Conclusions

The present comprehensive real-world study demonstrates the favorable medium- to long-term effectiveness and safety of UST in CD patients, with sustained benefits extending beyond 5 years (maximum follow-up 6.1 years; *n* = 43 at 5 years). The identification of albumin and CRP levels at week 8 as early predictive biomarkers provides a practical framework for treatment optimization, though external validation is warranted. The effectiveness of UST on luminal, perianal, and extraintestinal manifestations, combined with its favorable safety profile, supports its role as a versatile therapeutic option in CD management. These findings contribute valuable evidence for positioning UST within evolving treatment algorithms and highlight the importance of early biomarker assessment in achieving optimal long-term outcomes. Further long-term prospective multicenter studies are warranted to confirm these findings.

## Supplementary Material

otag059_Supplementary_Data

## Data Availability

Data are not publicly available.
