# Supplementary material for: Long-term drug persistence and clinical outcomes of ustekinumab in Crohn’s disease: a multicenter real-world study from the Hokkaido phoenix cohort
Source: Crohns Colitis 360. 2026 Jun 19;8(2):otag059. doi: 10.1093/crocol/otag059 (PMC13318491; doi:10.1093/crocol/otag059)
Supplement: otag059_Supplementary_Data [file otag059_supplementary_data.zip › Supplementary_Materials.docx]

**Supplementary Table S1.** **UST dose-interval modification details**

*Dose-interval modifications occurred in 22 of 317 patients (6.9%). Decisions were at the discretion of the treating physician, based on a combination of clinical symptoms, biomarker trends (CRP, albumin), endoscopic activity when available, and patient preference.*

| Modification pattern | n | Indication |
| --- | --- | --- |
| 12 → 8 weeks (intensification) | 10 | Inadequate clinical / biomarker response |
| 8 → 12 weeks (de-intensification) | 9 | Sustained clinical and biochemical remission, patient preference |
| 8 → 12 → 8 weeks (re-intensification) | 2 | Recurrence after de-intensification |
| 12 → 10 weeks | 1 | Mild biochemical worsening |
| **Total with any interval modification** | **22** |  |

Three-year UST persistence in the 22 modified patients was approximately 73%, comparable to 70% in non-modified patients (log-rank p = 0.78). The Japanese label for UST does not allow dosing intervals shorter than 8 weeks, so further intensification beyond every-8-week dosing was not possible*.*

**Supplementary Table S2.** **Baseline characteristics of the post-surgical reset subgroup vs. the overall cohort**

Comparison of the 17 patients who initiated UST primarily for post-operative remission maintenance ("post-surgical reset", induction reason = post-operative reset) with the overall study cohort.

| **Characteristic** | **Post-surgical reset (n=17)** | **Overall cohort (n=317)** |
| --- | --- | --- |
| Age, median (years) | 40 | 38 |
| Male sex, n (%) | 9/17 (53) | 200/317 (63) |
| Disease duration, median (years) | 8.7 | 10.9 |
| Baseline CDAI, median | 107 | 136 |
| Baseline albumin, median (g/dL) | 3.80 | 3.70 |
| Baseline CRP, median (mg/dL) | 0.18 | 0.31 |
| Bio-naïve, n (%) | 6/17 (35) | 65/317 (20.5) |
| Prior bowel resection, n (%) | 16/17 (94) | 161/317 (50.8) |
| Active perianal fistula at induction, n (%) | 1/17 (6) | 60/317 (18.9) |
| Median follow-up (days) | 1,202 | 829 |
| UST continuation, 1 / 3 / 5 yr (%) | 100 / 93.3 / 93.3 | 87.6 / 69.6 / 66.8 |

The post-surgical reset subgroup had substantially lower baseline disease activity than the overall cohort (lower CDAI, higher albumin, lower CRP) and almost all had prior bowel resection (94% vs 51%). These features support the interpretation that the apparently higher persistence rate reflects a reduced inflammatory burden after recent surgery and selection bias toward patients with quiescent disease, rather than a differential therapeutic effect of UST.

**Supplementary Table S3.** **UST persistence stratified by concomitant therapy at induction**

Kaplan–Meier UST continuation rates stratified by the presence of each concomitant therapy at the time of UST initiation. Log-rank tests compared each "yes" subgroup with its corresponding "no" subgroup.

| **Concomitant therapy** | **n** | **1-yr (%)**  **(95% CI)** | **3-yr (%)**  **(95% CI)** | **5-yr (%)**  **(95% CI)** | **log-rank**  **p** |
| --- | --- | --- | --- | --- | --- |
| Steroid (Yes) | 120 | 86.8  (79.1–91.9) | 65.4  (55.1–73.9) | 60.6  (49.6–70.0) | — |
| Steroid (No) | 197 | 88.1  (82.5–92.0) | 72.4  (64.5–78.8) | 70.8  (62.4–77.7) | 0.22 |
| Immunomodulator (Yes) | 128 | 88.6  (81.5–93.1) | 73.8  (64.3–81.1) | 69.4  (59.1–77.6) | — |
| Immunomodulator (No) | 189 | 87.0  (81.0–91.1) | 66.5  (58.0–73.7) | 65.3  (56.6–72.7) | 0.49 |
| Enteral nutrition ≥900 kcal (Yes) | 91 | 90.9  (82.6–95.4) | 68.2  (56.1–77.6) | 63.4  (50.3–74.0) | — |
| Enteral nutrition ≥900 kcal (No) | 226 | 86.3  (80.8–90.2) | 70.3  (62.9–76.5) | 68.3  (60.6–74.9) | 0.92 |

UST persistence did not differ significantly by any concomitant therapy. As all 120 patients on steroids at induction discontinued steroids within 1 year (per original Results section), the multi-year continuation rates can be interpreted as steroid-free UST persistence.

**Supplementary Table S4. Endoscopic remission – ulcer-absence (primary) vs. SES-CD ≤2 (sensitivity)**

Comparison of the primary endoscopic-remission endpoint (absence of all active inflammatory lesions: aphthae, erosions, and ulcers — corresponding to the ulcer-related sub-score = 0 of the SES-CD) with the SES-CD ≤2 composite cutoff that was requested as a sensitivity analysis. The ulcer-absence criterion is consistent with the endoscopic-healing target endorsed by the STRIDE-II treat-to-target consensus; the SES-CD ≤2 cutoff additionally requires resolution of the narrowing/stenosis sub-score, which scores fibrotic non-inflammatory strictures and therefore systematically misclassifies patients with healed inflammation but residual fibrostenotic complications. All patients with both a documented ulcer-absence assessment and a numeric SES-CD score recorded at the same time point are included.

| **Time** | **N** | **Ulcer-absence (primary) n (%)** | **SES-CD ≤2 (sensitivity) n (%)** | **Agreement (κ)** | **Median SES-CD (IQR)** |
| --- | --- | --- | --- | --- | --- |
| Baseline | 207 | 19 (9.2) | 22 (10.6) | 95.7% (κ=0.76) | 10 (6–16) |
| Week 52 | 130 | 28 (21.5) | 21 (16.2) | 85.4% (κ=0.52) | 6 (3–11) |
| Week 104 | 75 | 23 (30.7) | 19 (25.3) | 92.0% (κ=0.80) | 5 (3–10) |
| Week 156 | 49 | 13 (26.5) | 11 (22.4) | 91.8% (κ=0.78) | 6 (3–9) |

κ = Cohen's kappa coefficient. The two definitions yielded broadly concordant results (moderate to substantial agreement; κ 0.52–0.80). Both definitions show the same temporal pattern of progressive improvement under UST therapy. Numerical gaps between the two definitions (e.g., 21.5% vs 16.2% at week 52) are concentrated in patients who achieved complete resolution of all active inflammatory lesions but had a residual fibrotic stricture, demonstrating the sensitivity of the SES-CD ≤2 composite cutoff to the narrowing sub-score.

Note: the denominator in this table (N=207 at baseline) differs from that in Figure 3a (N=239) because Supplementary Table S4 includes only patients in whom both an ulcer-absence assessment and a numeric SES-CD score were recorded at the same visit; patients with ulcer-absence assessment but without a numeric SES-CD score (n=32 at baseline) are included in Figure 3a but not in this table.

**Supplementary Table S5. Adverse events.**

| Adverse event (34 patients) | Events, n |
| --- | --- |
| Any adverse event | 40 |
| Any adverse event at the subcutaneous injection site | 8 |
| General fatigue | 8 |
| Infections | 7 |
| Abdominal pain | 5 |
| Worsening of arthralgia | 3 |
| Malignancy | 2 |

Only cases involving two or more people and excluding exacerbation of Crohn’s disease are listed. Duplicate cases are also included.

The two malignancy cases listed represent newly diagnosed malignancies in patients without a prior cancer history.

**Supplementary Table S6. Drug-specific continuation rates of subsequent advanced therapies after UST discontinuation**

Continuation rates of each subsequent advanced therapy among the 75 patients receiving any subsequent advanced therapy after UST discontinuation. Class-stratified summaries are shown in bold rows.

| **Subsequent therapy** | **n** | **1-yr (%)**  **(95% CI)** | **2-yr (%)**  **(95% CI)** | **3-yr (%)**  **(95% CI)** |
| --- | --- | --- | --- | --- |
| Vedolizumab | 29 | 71.7 (51.3–84.7) | 44.8 (25.7–62.3) | 30.6 (13.7–49.3) |
| Adalimumab | 23 | 73.9 (50.9–87.3) | 73.9 (50.9–87.3) | 40.7 (13.7–66.6) |
| Infliximab | 17 | 47.1 (23.0–68.0) | 29.4 (10.7–51.1) | 11.0 (0.9–35.5) |
| Risankizumab | 5 | 100 | 100 | — |
| Upadacitinib | 1 | — | — | — |
| Class: anti-TNF (IFX+ADA) | 40 | 62.5 | 54.7 | 27.9 |
| Class: anti-integrin (VDZ) | 29 | 71.7 | 44.8 | 30.6 |
| Class: IL-23p19 / JAK (RZB+UPA) | 6 | 83.3 | 83.3 | 83.3 (small n) |

Numbers do not sum to the 86 UST discontinuations because patients without subsequent treatment, those enrolled in clinical trials, and those switched to other non-advanced therapies were excluded from this analysis.

The 1- and 2-year continuation rates for adalimumab are identical (73.9%), reflecting no discontinuation events between 12 and 24 months in this subgroup.

RZB and UPA were approved in Japan in 2023. Individual drug-level 3-year estimates are not reported given the very small sample sizes (RZB n=5, UPA n=1). The combined class 3-year rate of 83.3% is based on the subset of patients with ≥3 years of follow-up and should be interpreted with caution (CI uninformative).

**Supplementary Table S7. Sensitivity analysis – UST continuation in patients enrolled after the recruitment window of our 2020 publication**

Cohort split based on UST initiation date, to assess robustness of the primary outcome to potential overlap with our 2020 publication (Ito et al., Crohn's Colitis 360. 2020;2:otaa061; recruitment June 2017 – September 2019, n=137 patients shared with the present cohort).

| **Cohort** | **n** | **1-year (95% CI)** | **3-year (95% CI)** | **5-year (95% CI)** |
| --- | --- | --- | --- | --- |
| Full cohort | 317 | 87.6 (83.3–90.9) | 69.6 (63.5–75.0) | 66.8 (60.2–72.5) |
| Pre-October 2019 (overlap window) | 153 | 87.4 (80.9–91.8) | 72.4 (64.4–78.9) | 69.3 (61.1–76.1) |
| Post-October 2019 (new only) | 164 | 87.8 (81.3–92.1) | 63.2 (51.1–73.0) | 63.2 (51.1–73.0) |

Log-rank test between 'pre-October 2019' and 'post-October 2019' subgroups: p = 0.34. Of the 317 patients, 137 (43.2%) overlap with our 2020 publication (Ito et al. 2020); 180 (56.8%) — comprising 16 from an additional 7th institution and 164 newly enrolled after September 2019 — are reported here for the first time. In a multivariate Cox model restricted to the post-October 2019 subgroup (n=164), the week-8 biomarker predictors retained statistical significance: week-8 albumin ≥3.3 g/dL HR 0.36 (95% CI 0.16–0.81, p=0.013); week-8 CRP ≥0.33 mg/dL HR 1.94 (95% CI 1.05–3.59, p=0.034).

**Supplementary Figure S1.** **Univariate analysis of factors associated with UST continuation.**


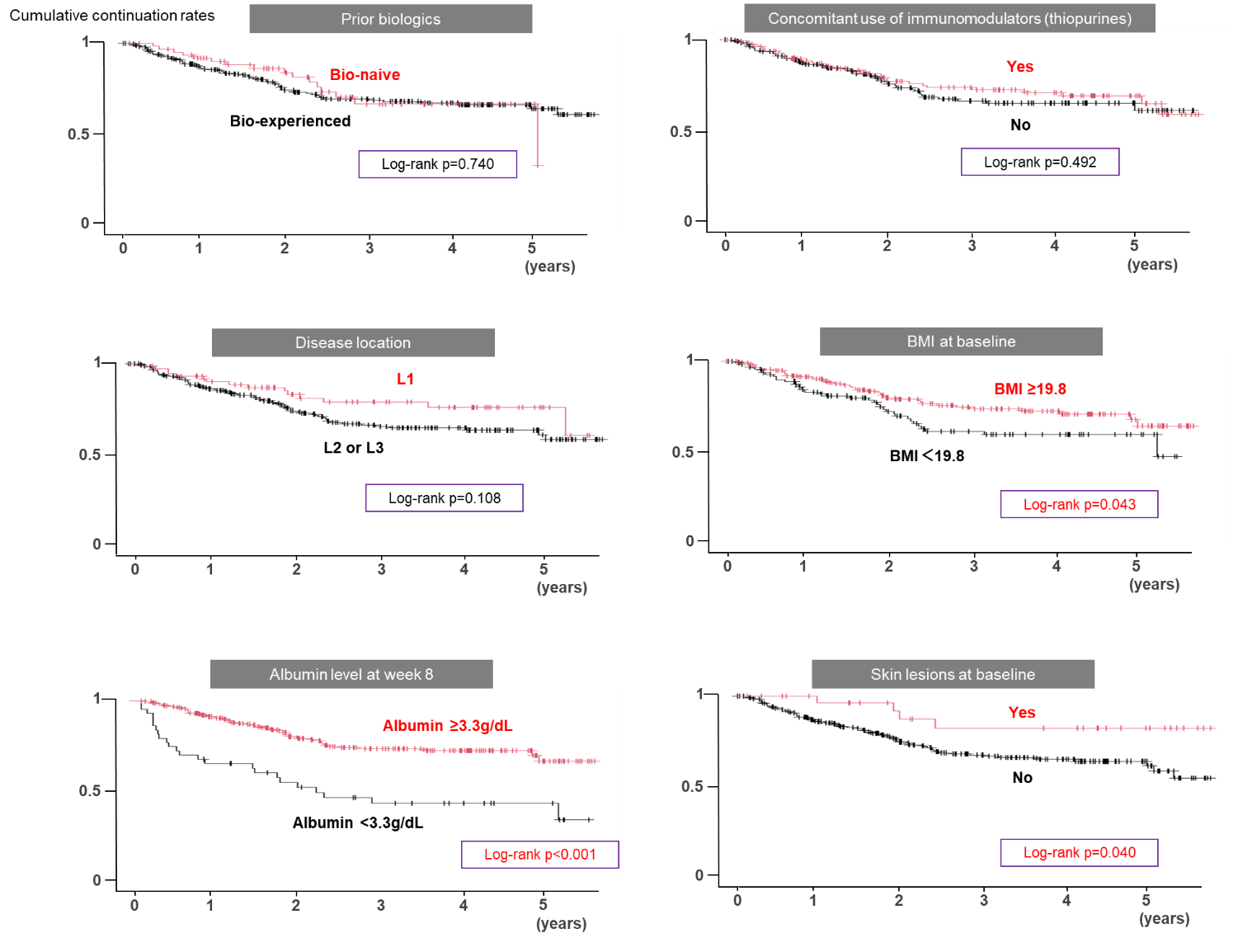


The Kaplan–Meier curve illustrates the UST continuation rates based on the various baseline and week 8 parameters associated with these rates in the univariate analysis. UST, ustekinumab.

**Supplementary Figure S2. Kaplan–Meier curves for UST continuation: post-surgical reset vs. other indications**


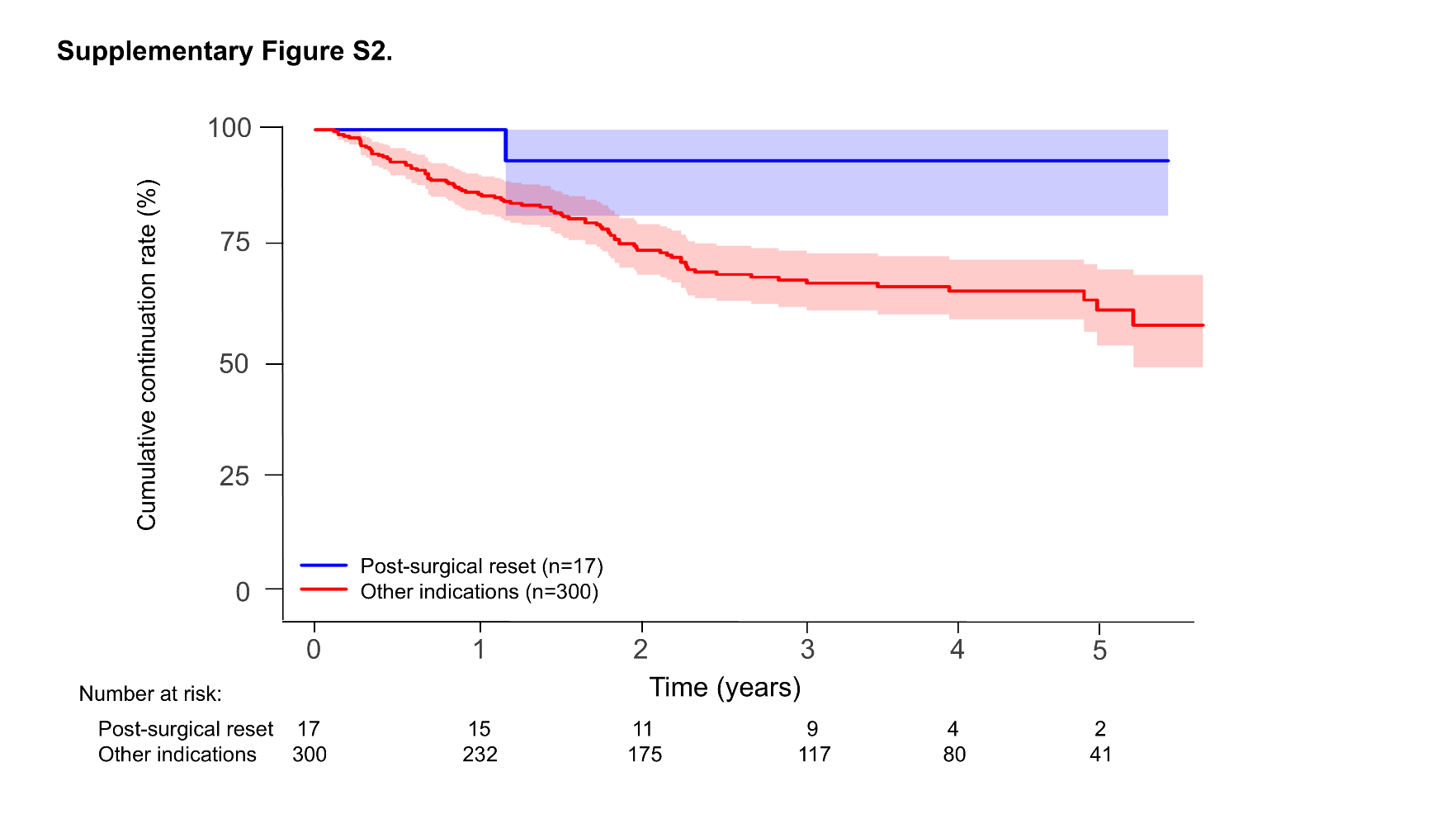


Kaplan–Meier curves (with 95% confidence intervals) of UST continuation among 17 patients initiated for post-operative remission maintenance ("post-surgical reset") versus 300 patients initiated for other indications. The 1-, 3-, and 5-year continuation rates in the post-surgical subgroup were 100%, 93.3%, and 93.3% respectively. As shown in Supplementary Table S2, the post-surgical subgroup had markedly lower baseline disease activity than the overall cohort, supporting the interpretation that this difference reflects selection bias toward quiescent disease rather than a differential therapeutic effect of UST. UST, ustekinumab.

**Supplementary Figure S3. Kaplan–Meier curves – drug-class–stratified continuation of subsequent advanced therapies after UST discontinuation**


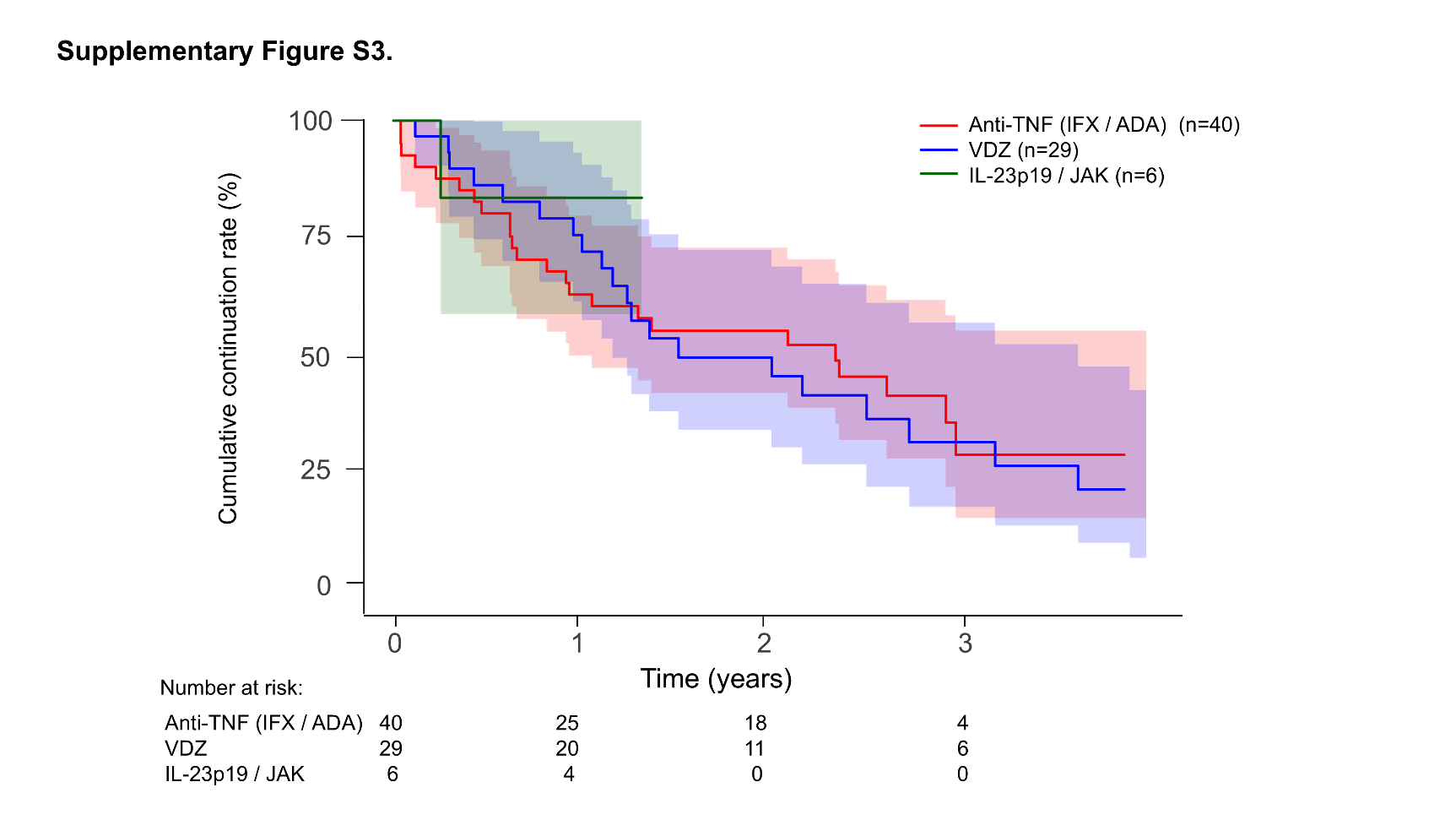


Kaplan–Meier curves stratified by drug class of the subsequent advanced therapy initiated after UST discontinuation: anti-TNF (IFX or ADA, n = 40), anti-integrin (VDZ, n = 29), and IL-23p19 / JAK inhibitors (RZB or UPA, n = 6). The 3-year continuation rates were 27.9% (anti-TNF), 30.6% (VDZ), and 83.3% (IL-23p19 / JAK; based on a small subgroup, CI uninformative). These exploratory comparisons should be interpreted with caution given the differing indications for each subsequent therapy and the small numbers in the IL-23p19 / JAK arm.
